# Supplementary material for: Chemical Characterization and Anti-HIV-1 Activity Assessment of Iridoids and Flavonols from Scrophularia trifoliata
Source: Molecules. 2021 Aug 6;26(16):4777. doi: 10.3390/molecules26164777 (PMC8398805; doi:10.3390/molecules26164777)

# Supplementary Materials

# Chemical Characterization and Anti-HIV-1 Activity Assessment of Iridoids and Flavonols from *Scrophularia trifoliata*

**Francesca Guzzo [1], Rosita Russo [1], Cinzia Sanna [2], Odeta Celaj [1], Alessia Caredda [3], Angela Corona [3], Enzo Tramontano [3], Antonio Fiorentino [1,4], Francesca Esposito [3,*] and Brigida D'Abrosca [1,4,*]**

[1] Department of Environmental Biological and Pharmaceutical Sciences and Technologies, DiSTABiF University of Campania Luigi Vanvitelli, Via Vivaldi 43, 81100 Caserta, Italy;
francesca.guzzo@unicampania.it (F.G.); rosita.russo@unicampania.it (R.R.);
odeta.celaj@unicampania.it (O.C.); antonio.fiorentino@unicampania.it (A.F.)

[2] Department of Life and Environmental Sciences, University of Cagliari, Via Sant'Ignazio da Laconi 13, 09123 Cagliari, Italy; cinziasanna@unica.it

[3] Department of Life and Environmental Sciences, University of Cagliari, Cittadella Universitaria di Monserrato, ss554, km 4500, Monserrato, 09042 Cagliari, Italy;
alessiacaredda@unica.it (A.C.);
angela.corona@unica.it (A.C.); tramon@unica.it (E.T.)

[4] Department of Marine Biotechnologies, Stazione Zoologica Anton Dohrn, Villa Comunale, 80121 Naples, Italy

* Correspondence: francescaesposito@unica.it (F.E.); brigida.dabrosca@unicampania.it (B.D.)

**Supplementary figure S1.** CIGAR-HMBC experiment of compound **5**

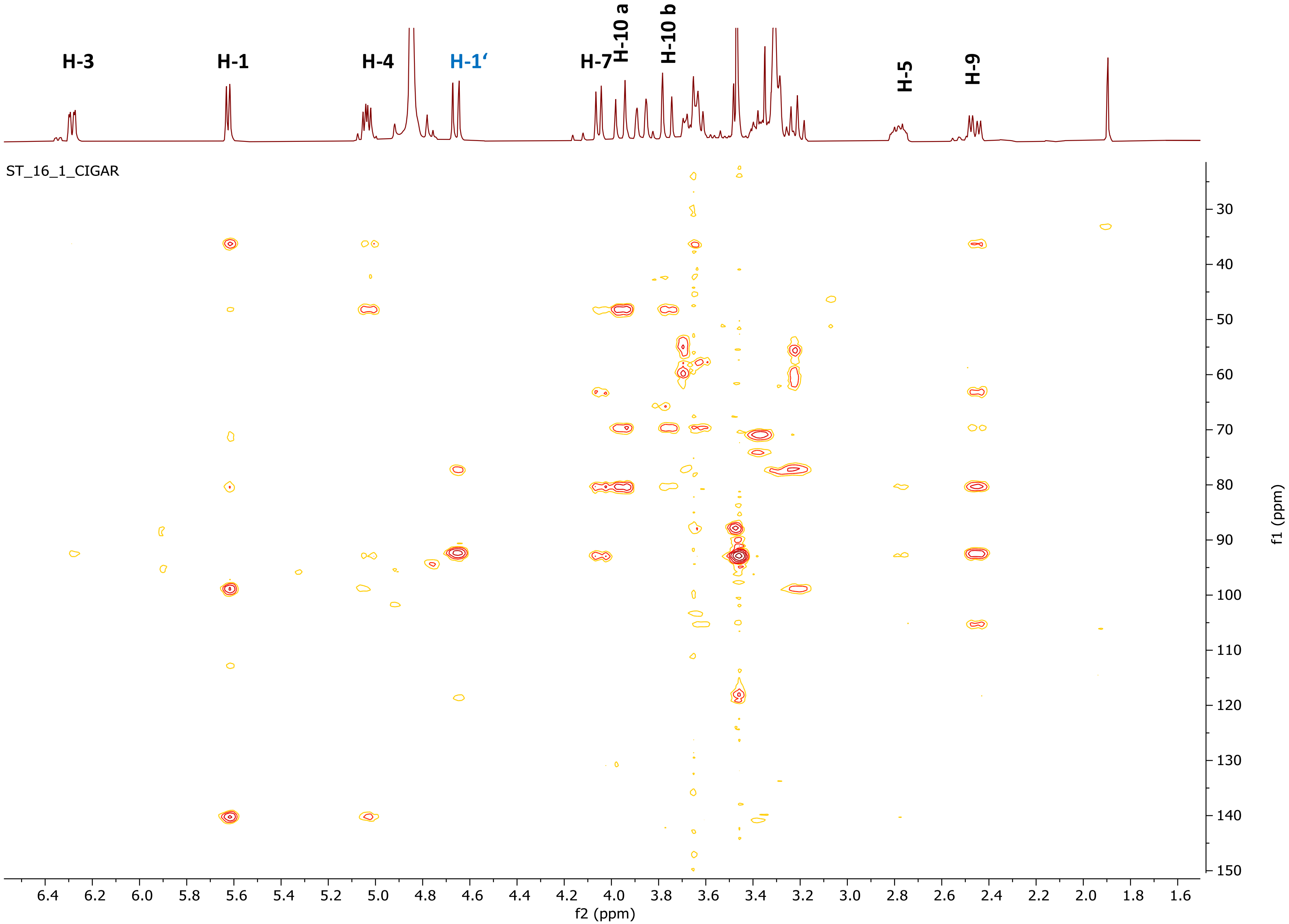

**Supplementary figure S2.** Key nOe correlation of compound **5** (top); NOESY experiment of compound **5** in $CD_3OD$ (bottom).

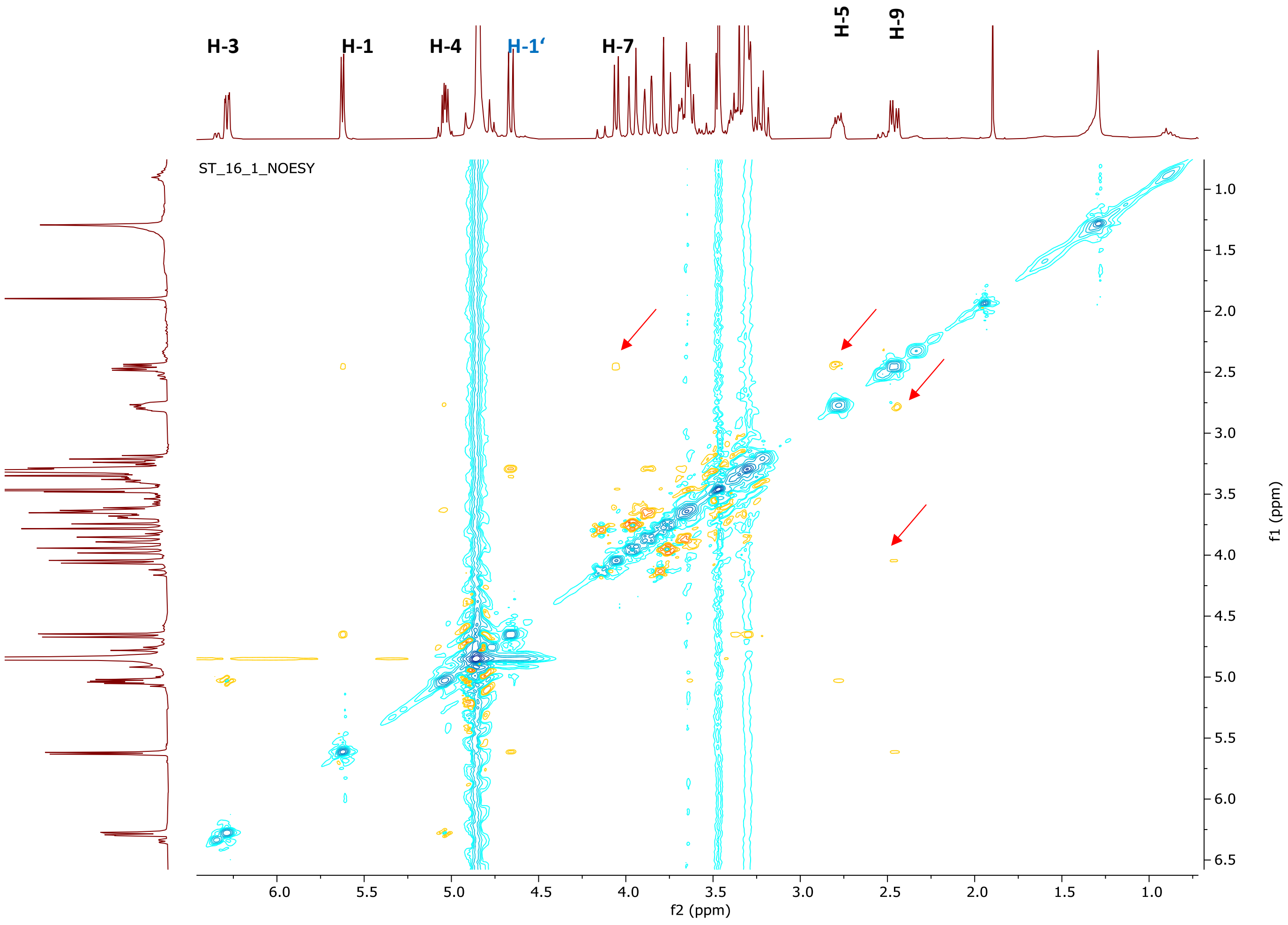

**Supplementary figure S3.** HSQC experiment of compound ***13***

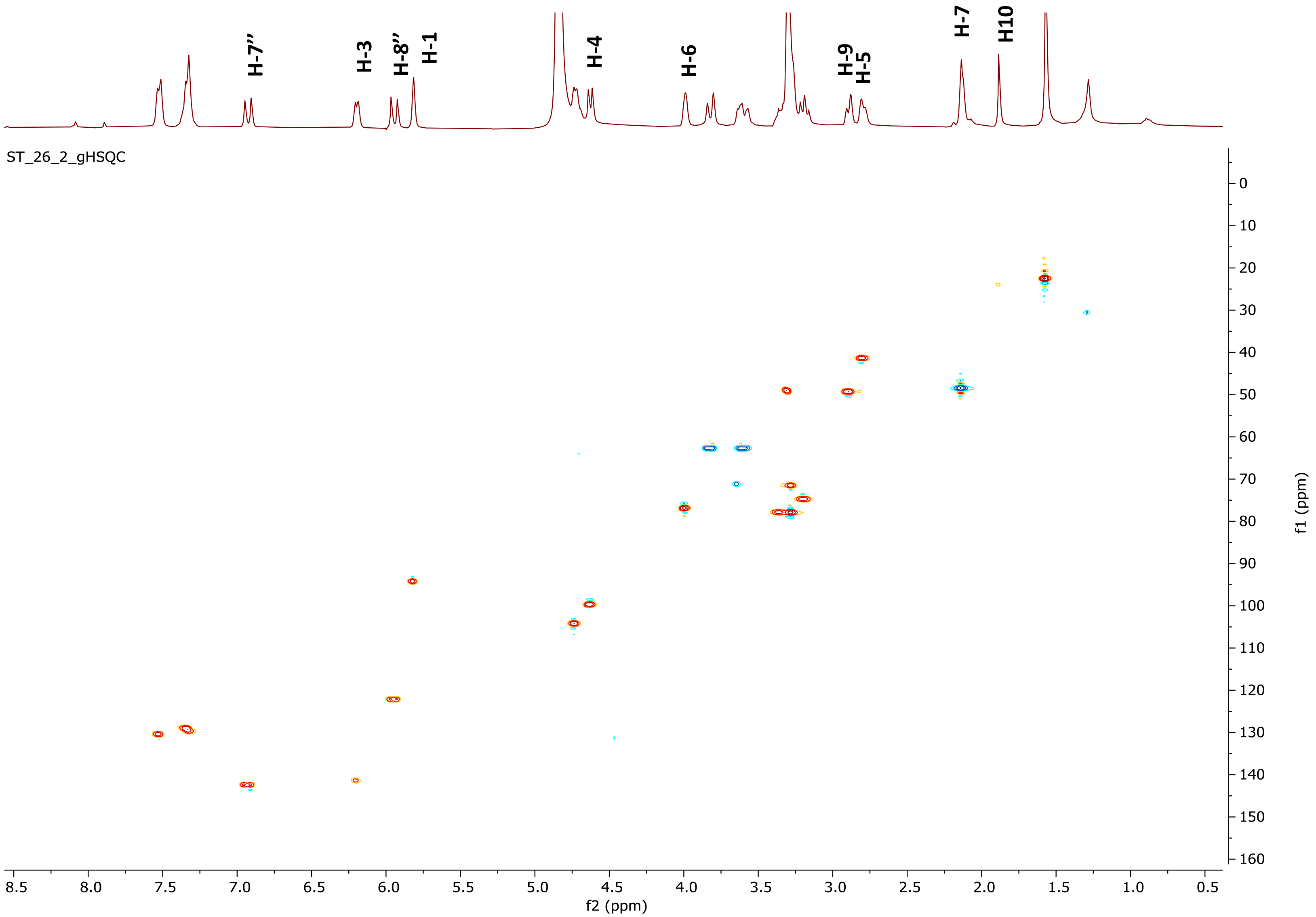

Supplement: Supplementary file 1 [file molecules-26-04777-s001.zip › molecules-1322673-supplementary.pdf]
